# Multiple ALMT subunits combine to form functional anion channels: A case study for rice ALMT7

**DOI:** 10.3389/fpls.2022.1012578

**Published:** 2022-11-14

**Authors:** Hui Zhou, Zhuoran Hu, Yunxin Luo, Cuizhu Feng, Yu Long

**Affiliations:** State Key Laboratory of Crop Stress Adaptation and Improvement, School of Life Sciences, Henan University, Kaifeng, China

**Keywords:** ALMT7, malate flux, transmembrane helices, homomer, channel activity

## Abstract

The Aluminum Activated Malate Transporter (ALMT) family members are anion channels that play important roles in organic acid transport, stress resistance, growth, development, fertilization and GABA responses. The rice malate permeable OsALMT7 influences panicle development and grain yield. A truncated *OsALMT7* mutant, *panicle apical abortion1* (*paab1)* lacking at least 2 transmembrane helices, mediates reduced malate efflux resulting in yield reducing. Here, we further investigated the contribution of OsALMT7 transmembrane helices to channel activity, using heterologous expression in *Xenopus laevis* oocytes. We further found that OsALMT7 formed as a homomer by co-expressing OsALMT7 and paab1 proteins in oocytes and detecting the physical interaction between two OsALMT7, and between OsALMT7 and paab1 mutant protein. Further study proved that not just OsALMT7, mutants of TaALMT1 inhibit wild-type TaALMT1 channel, indicating that ALMTs might perform channel function as homomers. Our discovery brings a light for ion channel structure and homomultimer regulation understanding for ALMT anion channels and potential for crop grain yield and stress response improvement in the context of the essential role of ALMTs in these plant processes.

## Introduction

Aluminum Activated Malate Transporters (ALMTs) are important plant anion channels by playing roles in organic acid transport, stress resistance, growth and development, nutrient absorption, and the GABA response (Hedrich, 2012; Ramesh et al., 2015; Sharma et al., 2016; Balzergue et al., 2017; Medeiros et al., 2018; Long et al., 2020). Wheat TaALMT1 was the first identified family member (named due to its activation by aluminium (Al^3+^), the main ion causing toxicity to plants in acidic soil) mediates malate efflux to chelate Al^3+^ and relieve toxicity (Zhang et al., 2001; Sasaki et al., 2004). Our recent work has shown that TaALMT1 not only participates in Al^3+^ detoxification but also participates in the GABA response (Ramesh et al., 2015; Long et al., 2020). *Arabidopsis* has 14 ALMTs, with those characterized having varied roles, and several others yet to be characterized (Hedrich, 2012; Sharma et al., 2016; Medeiros et al., 2018). AtALMT1, which is mainly expressed in roots and localized at the plasma membrane (PM), functions in Al^3+^ toxicity resistance, similarly to TaALMT1, low phosphorus (LP) signal transduction, and Fe uptake, by mediating malate efflux in roots (Hoekenga et al., 2006; Balzergue et al., 2017; Mora-Macías et al., 2017). ALMT3 is also involved in the LP response in root hairs (Maruyama et al., 2019). The vacuolar membrane (tonoplast)-localized anion channels ALMT4, ALMT6, and ALMT9 transport anions across the tonoplast to regulate stomatal movement and/or salinity stress resistance (Kovermann et al., 2007; Meyer et al., 2011; De Angeli et al., 2013a; Baetz et al., 2016; Eisenach et al., 2017). ALMT12, ALMT13, and ALMT14, are all likely to be R-type anion channels in the PM, playing roles in malate transport to regulate stomatal opening, stomatal and mesophyll conductance, and pollen tube growth (Meyer et al., 2010; Sasaki et al., 2010; Medeiros et al., 2016; Gutermuth et al., 2018; Domingos et al., 2019). Furthermore, ALMT family members were reported to be involved in physiological and stress response processes in other crops and plants including maize, rice, soybean, apple, grape, tomato, canola, *Brachypodium distachyon*, and *Medicago sativa* (Ligaba et al., 2006; Piñeros et al., 2008; Chen et al., 2013; De Angeli et al., 2013b; Sasaki et al., 2016; Sharma et al., 2016; Liu et al., 2017; Peng et al., 2018; Heng et al., 2018; Li et al., 2020; Luu et al., 2019).

Previously, through the use of gene mapping, a mutation in OsALMT7 was found to underpin a panicle apical abortion (*paab1*) phenotype. The two *paab1* transcripts (*paab1-t1* and *paab1-t2*), were found to be transcribed into proteins with C-terminal truncations (containing 163 amino acid residues) that transport anions with a capacity lower than that of the full-length OsALMT7 (Heng et al., 2018). OsALMT7 (paab1) is expressed in vascular tissues of roots, stems, sheaths, and panicles and localizes to the PM, and it was concluded that it plays an important role in panicle anion transport, panicle development, and grain yield (Heng et al., 2018). Furthermore when wild-type plants were transformed with a genomic fragment containing the *paab1* base substitution resulting in a panicle abortion phenotype (Heng et al., 2018). Compare with other ALMTs, OsALMT7 has special molecular characteristicses that it has the ability to mediate malate flux with its transmembrane helices incomplete, and its truncated protein represses the wild-type channel in rice. Here, we investigate the mechanism of these characteristicses with further electrophysiology studies. We proposed and tested the hypothesis that OsALMT7 transports malate as a multimer and that paab1 interaction with OsALMT7 inhibits transport capacity. Furthermore, we extended this to examine whether this is a feature of other ALMTs, specifically TaALMT1.

A number of plant channels are already known to function as homo- or hetero-multimers, and heteromerization is acknowledged to be an important mechanism of channel regulation (Hedrich, 2012). For example, aquaporins of both the PM and tonoplast assemble as homo- or heterotetramers, such as PIP1s with no or weak water permeability with itself, combine with PIP2s and increase their activity in different plant species (Harvengt et al., 2000; Bienert et al., 2012; Fetter et al., 2004; Heinen et al., 2014; Berny et al., 2016). Shaker family potassium (K) channels share a similar structure with ALMTs, 6 transmembrane α-helices with both N-terminal and C-terminal cytosolic domains, and function as homo- or heterotetramers; notably, KC1, the silent channel subunit, forms a heterotetramer with other shaker K channels to change their voltage dependence (Baizabal-Aguirre et al., 1999; Pilot et al., 2001; Véry and Sentenac, 2003; Xicluna et al., 2007; Duby et al., 2008; Lebaudy et al., 2008; Geiger et al., 2009; Lebaudy et al., 2010; Wang et al., 2010; Jeanguenin et al., 2011). Cyclic nucleotide-gated channel 2 (CNGC2) and CNGC4 have been proposed to form a heteromeric channel to mediate Ca^2+^ currents (Tian et al., 2019). For plant anion channels, the S-type channel SLAC1 has been proposed to combine with the shaker K^+^ channels KAT1 and KAT2 and inhibit their channel activity (Zhang et al., 2016). Zhang et al. (2013) showed that tonoplast-localized AtALMT9 formed homomultimeric complex by coexpressing the mutant channel and wild-type channel in tobacco leaves; however, it has not been determined whether PM-localized ALMTs, including OsALMT7, and R-type anion channels, such as ALMT12/13/14, function as multimers or monomers.

Here, we determined that OsALMT7 function as multimeric proteins and that combinations of ALMT subunits can contribute to anion channel regulation. This strengthens our understanding of ALMT function. By defining the mechanism by which subunit modification has a dominant effect on channel function, this provides a new avenue by which genetic modification or gene editing can have important effects without first creating knockout mutants, enabling crop stress resistance and grain yield improvements.

## Materials and methods

### Electrophysiological measurements in *X. laevis* oocytes

All chemicals were sourced from Sigma Aldrich. Capped complementary RNA (cRNA) production, *X. laevis* oocytes preparation, and whole oocyte two-electrode voltage clamping (TEVC) recording were performed as our previous work (Heng et al., 2018; Long et al., 2020). 46 nL of 200 mM Na_2_-malate (pH 7.2) was pre-loaded before TEVC recording. The bath solution for malate current recording consisted of 80 mM Na-gluconate, 1 mM Ca-gluconate_2_, 1 mM K-gluconate, 1 mM Mg-gluconate_2_, 25 mM malic acid, 0.1 mM LaCl_3_, and 10 mM MES/Tris (pH 5.8). And the bath solution for different anion permeability contained 25 mM NaNO_3_, 25 mM Na_2_-malate, 25 mM NaCl, and 25 mM Na_2_SO_4_ respectively. The method for creating inside-out patch clamp in *X. laevis* oocytes followed our previous protocols (Long et al., 2020) with the solution modified, 20 mM Na_2_-malate in the bath solution (cytosolic side) and 10 mM Na_2_-malate in the pipette solution (outside) and the pH of both was adjusted to 7.2 (Hepes/Tris).

### BiFC assay

The coding regions of *OsALMT7*, *paab1-t1*, *paab1-t2*, and *TaALMT1* were cloned into pSPYCE and pSPYNE vectors (Waadt et al., 2008; Waadt and Kudla, 2008). The BiFC assays were performed as described previously (Walter et al., 2004; Waadt and Kudla, 2008). The tested construct pairs were expressed in leaves of *Nicotiana benthamiana* for 3 d before microscopy observation. The YFP fluorescence in the transformed leaves was imaged using a confocal laser scanning microscope (ZEISS LSM710).

## Results

### Paab1 mutant proteins lacking the last 2-3 transmembrane helices mediate malate efflux

**Figure 1:**
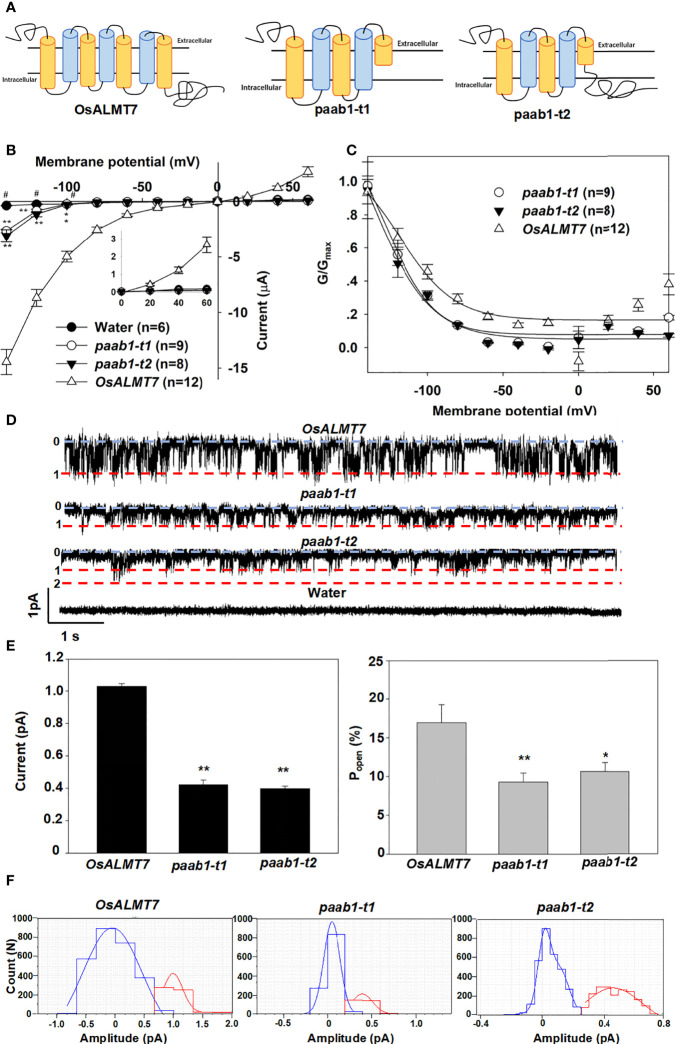
OsALMT7 truncate mutant proteins (paab1-t1 and paab1-t2) showed channel activity in X *lavies* oocytes. **(A)** Schematic of OsALMT7, paab1-t1, and paab1-t2. **(B)** Current-Voltage relationship from TEVC recordings of whole X *laevis* oocytes expressing *OsALMTl, paab1-t1, paab1-t2*, and water injected control with 46 nol f 200 mM Na 2-malate preloaded. The data are derived from the recordings shown in **(A)** and presented as mean ± SE. Student's t test was used to analyze statistical significance from water injected control (*P<0.1 and **P<0.01). **(C)** G/Gmax-Voltage relationship from *OsALMT1, paab1-t1*, and *paab1-t2* expressed oocytes. The data are derived from the recordings shown in **(A)**. **(D)** Channel activity of representative inside-out membrane patch from *OsALMT1, paab1-t1*, and *paab1-t2* expressed oocytes with 20 mM malate in the bath (equivalent to the cytosol) and 10 mM malate in the pipette (equivalent to the cell exterior) both at pH 7.2, at a holding voltage equivalent to -160 mV in the whole cell configuration. Downward current deflections are indicative of anion efflux from the cell. The numbering, 0 (and blue line) indicates channel closure and 1, 2 indicate number of channels simultaneously open (red lines). **(E)** The single channel current (left panel) and open probability (right panel) on *OsALMTl, paab1-t1*, and *paab1-t2* expression oocytes. The data are derived from the recordings shown in **(D)** and additional data and presented as means± SE (n>10 for each data). Student's t test was used to analyze statistical significance from control conditions (**P<0.01). **(F)** Histogram analysis of OsALMT7, paab1-t1, and paab1-t2 induced currents from inside-out recording blue and red lines indicate Gaussian fits using Clampfit. This and additional data were used to generate **(D)**.

Heng et al. (2018) found two transcripts in a panicle apical abortion (*paab1*) mutant rice, *paab1-t1* and *paab1-t2*. **Figure 1A** depicts the full-length OsALMT7 protein and the paab-t1 and paab1-t2 variants in which the last 2-3 transmembrane α-helices (depending on topology predictions) and the cytosolic C-terminus are absent. Both paab1 truncations encode malate-transport competent proteins but with a much-reduced capacity compared to full-length OsALMT7 when expressed in *X. laevis* oocytes (Heng et al., 2018). Here, we carried out an in-depth analysis of the behaviour of paab1 proteins in *X. laevis* oocytes. Our results confirmed that the paab1-t1 and paab1-t2 proteins conducted significantly greater inward currents than water-injected oocytes at the polarization range of membrane potential (more negative than -100 mV), but these currents were lower in magnitude than those of wild-type OsALMT7 (malate efflux; **Figure 1B**; Supporting **Figure 1**). Channel conductance (G) analysis of the paab1 mutant truncated proteins found that the maximal conductance was lower, and voltage-dependent activation occurred at a more negative potentials than that of the wild-type channel (OsALMT7). The G_max_ values of OsALMT7, paab1-t1, and paab1-t2 were 112.2 ± 13.1 μS, 20.7 ± 5.8 μS, and 16.7 ± 5.2 μS, and the V_1/2_ values of OsALMT7, paab1-t1, and paab1-t2 were -118.4 ± 11.9 mV, -136.9 ± 16.0 mV, and -138.1 ± 18.0 mV, respectively (**Figure 1C**). Isolated inside-out membrane patches (cytosolic side facing the bath solution) from *X. laevis* oocytes injected with *OsALMT7*, *paab1-t1*, or *paab1-t2* cRNA showed, compared to water-injected controls, weak activated currents with short open times reminiscent of TaALMT1 (≈1 pA; Long et al., 2020). paab1 mutants showed both a smaller magnitude of activated currents and less open probability (P_open_), with single channel currents at -160 mV of 1.03 ± 0.03 pA, 0.42 ± 0.05, and 0.40 ± 0.04 pA and P_open_ of 16.9 ± 3.8%, 9.3 ± 1.3%, and 10.5 ± 1.2% for OsALMT7, paab1-t1, and paab1-t2, respectively (**Figures 1D, E**).

### Paab1 mutant channels inhibit OsALMT7

**Figure 2:**
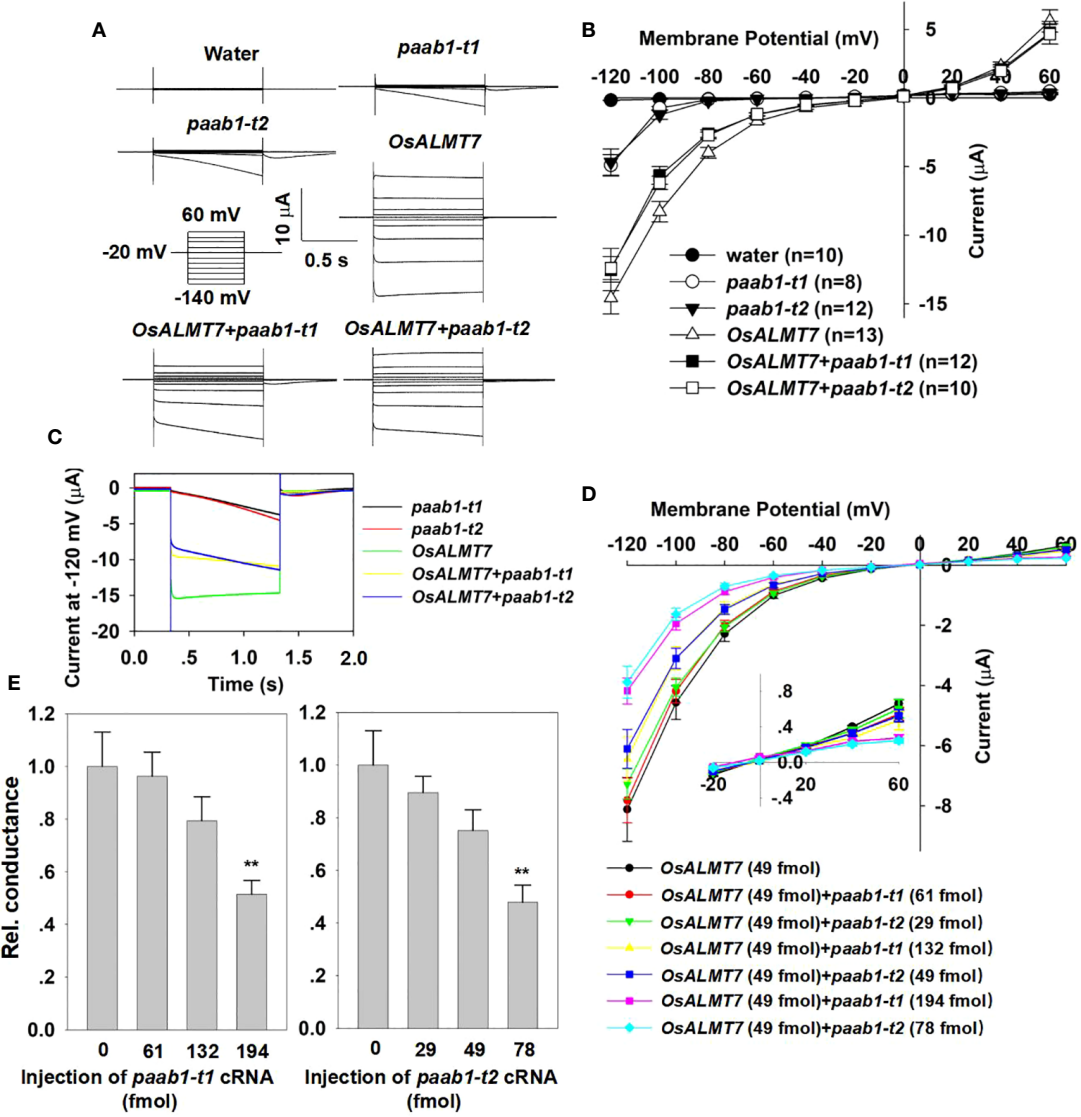
paab1-t1 and paab1-t2 inhibits channel activity of OsALMT7 in *X. lavies* oocytes. **(A)** TEVC current recording in *X. /aevis* oocytes. Whole-cell currents were recorded in oocytes injected with different cRNAs and cRNA combines: *OsALMTl, paab1-t1, paab1-t2, OsALMT1+paab1-t1, OsALMT1+paab1-t2*, and with water as control. Voltage protocols and time and current scale bars for the recordings are shown. **(B)** 1-V relationship of the currents recordings of oocytes expressing *OsALMT1, paab1-t1, paab1-t2, OsALMT7+paab1-t1, OsALMT7+paab1-t2*, and water injected control. The data are derived from the current recordings as shown in **(A)** and presented as mean ± SE. **(C)** Current of whole oocytes expression *OsALMTl, paab1-t1, paab1-t2, OsALMT1+paab1-t1*, and *OsALMT1+paab1-t2* at -120 mV **(D)** 1-V relationship of the currents recording of oocytes injected with different amount of *paab1* mutant cRNA co­injected with *OsALMTl .* The data are presented as mean ± SE (n;::12 for each data). **(E)** Rei. conductance of different amount of *paab1* mutant cRNAs injected oocytes. Student's t-test (**P<0.01) was used to analyze statistical significance.

Heng et al., 2018 showed that transgenic plants expressing both *OsALMT7* and *paab1* had a similar phenotype to *paab1-1* plants; we propose that this may be the result of paab1 mutant channels inhibiting OsALMT7 channel function. To investigate the impact of paab1 on OsALMT7 channel activity, we coexpressed *OsALMT7* and either of the two *paab1* transcripts. The TEVC recording showed that co-expression of either of the paab1-t1 and paab1-t2 proteins inhibited the channel activity of OsALMT7. Current magnitudes mediated by OsALMT7 and paab1 co-injection with identical amounts of cRNA fell between those obtained with sole injection of OsALMT7 or paab1 alone (**Figures 2A, B**). Interestingly, we found that OsALMT7 and paab1 mutant channels showed different time dependence, with OsALMT7 exhibiting instantaneous currents while paab1 currents had time-dependent activation at negative membrane potentials (**Figure 2A**). What about the current curve of *paab1* and *OsALMT7* coexpressing situation? As shown in **Figures 2A, C**, OsALMT7 and paab1 co-injection resulted in currents possessing both instantaneous and time-dependent components, taking on hybrid characteristics of both parent channels.

To confirm that paab1 inhibited OsALMT7, we injected different proportions of cRNA, increasing the cRNA amount of the *paab1* mutant to the same amount of OsALMT7 cRNA. As *paab1* cRNA was increased, the inhibition of OsALMT7 increased (**Figures 2D, E**). These TEVC results in *X. laevis* oocytes are consistent with the inhibition of OsALMT7 by paab1 mutant channels.

In Heng et al, we reported that OsALMT7 had a high permeability to NO_3_^-^ and malate, and a low permeability to Cl^-^ and SO_4_^2-^. In this study, we examined the anion permeability following paab1 and OsALMT7 co-injection by substitution of the anions in the bath solution. TEVC showed that paab1 mutant channels had no effect on anion permeability to OsALMT7; the currents following paab1-OsALMT7 co-injection shared the same anion selectivity as OsALMT7 (**Supplemental Figure 1**). We also investigated the effect of pH on OsALMT7, paab1-t1, paab1-t2, paab1-t1-OsALMT7, and paab1-t2-OsALMT7 channels, with the external bath pH setting to 7.2, 5.8, and 4.2, representing alkali, neutral, and acidic soil conditions. The TEVC recording showed that all these channels shared no dependence on external pH (**Supplemental Figure 2**), unlike wheat ALMT1 (Delhaize et al., 2004; Sasaki et al., 2004). In summary, paab1 did not affect the anion selectivity or pH dependence of OsALMT7.

### OsALMT7 functions as a multimer

**Figure 3:**
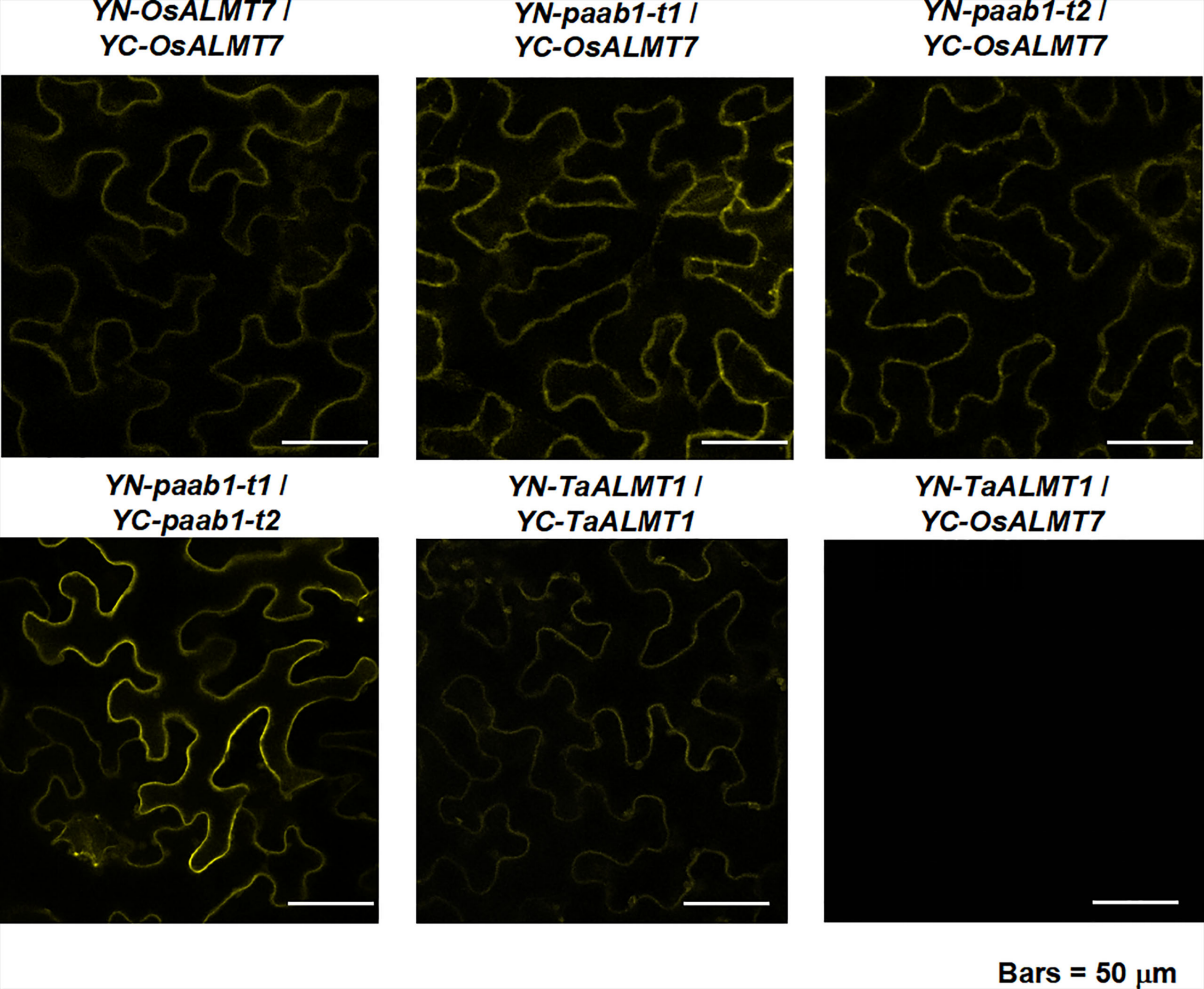
BiFC analysis between OsALMT7 and OsALMT7 (upper pannel, left image), OsALMT7 and paab1-t1 (upper pannel, middle image), OsALMT7 and paab1-t1 (upper pannel, right image), paab1- t1and paab1-t2 (lower pannelleft image), and TaALMT1 and TaALMT1 (lower pannel middle imgge). YN-TaALMT1 and YC-OsALMT7 was co-expressed as a negetive control.

We hypothesized that paab1 mutant channels inhibited OsALMT7 by combining into heteromers. To test our hypothesis, we first examined the physical interactions between OsALMT7 itself, OsALMT7 and paab1 mutant channels and the two paab1 mutant channels. BiFC experimental results were consistent with that OsALMT7 interacting with itself, paab1-t1, and paab1-t2 proteins in tobacco leaves and the two paab1 channels interacting with each other as well (**Figure 3**). TaALMT1 was used as a PM localized control and did not interact with OsALMT7, but interacted with itself (**Figure 3**).

**Figure 4:**
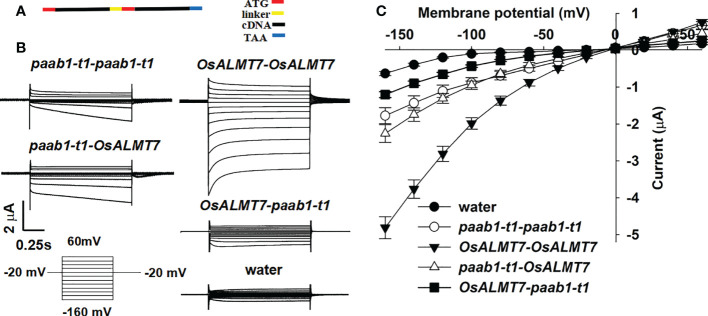
Properties of currents recorded in oocytes injected with different cRNAs encoding tandem subunits. **(A)** Diagram illustrating the construction of the tandems, stop code of the 5' teminal eDNA was removed and a linker encoding (GGGGSh was involved between the two subunits. The ATG of 3' teminal eDNA was arraied after the linker and the TAA was set at the end of the tandems. **(B)** TEVC current recording in *X. /aevis* oocytes. Whole-cell currents were recorded in oocytes injected with different tandem cRNAs: *paab1-t1-paab1-t1, OsALMT7-0sALMT7, paab1-t1-0sALMT7*, and *OsALMT1-paab1-t1.* Voltage protocols and time and current scale bars for the recordings are shown. **(C)** 1-V relationship of the currents recordings of oocytes expressing *paab1-t1-paab1-t1, OsALMT7-0sALMT7, paab1-t1-0sALMT7*, and *OsALMT1-paab1-t1.* The data are derived from the current recordings as shown in **(A)** and presented as mean ± SE.

Lebaudy et al. provided strong evidence that KAT1 and KAT2 formed heteromers by constructing *KAT1-KAT1*, *KAT1-KAT2*, *KAT2-KAT2*, and *KAT2-KAT1* tandems (translationally fused proteins) and expressing them in *X. laevis* oocytes. To confirm that paab1 mutant channels and OsALMT7 combined and formed a heteromer, tandems of *OsALMT7-OsALMT7*, *OsALMT7-paab1-t1*, *paab1-t1-paab1-t1*, and *paab1-t1-OsALMT7* were constructed and assayed for channel activity in *X. laevis* oocytes (**Figure 4A**). As shown in **Figure 4**, all the constructs were functional (had currents in excess of those of the water-injected controls); the OsALMT7-OsALMT7 tandem construct mediated the strongest malate currents, and the paab1-t1-paab1-t1 tandem construct mediated the weakest malate current. For the combination constructs, the tandem with OsALMT7 fused at the N-terminus had the weaker channel activity, while the tandem with paab1-t1 in the N-terminus showed stronger channel activity with an intermediate value between the *OsALMT7-OsALMT7* and *paab1-t1-paab-t1* constructs (**Figure 4**). Furthermore, *paab1-OsALMT7* showed stronger current in magnitude, while *OsALMT7-paab1* showed similar current shape in time dependent with *OsALMT7-OsALMT7*. We propose that these performances were caused with the artificial multimer with the way of tandem constructing. The TEVC recordings of these tandem constructs expressed in *X. laevis* oocytes provided further evidence that paab1 mutant channels and OsALMT7 assemble to form homo- or heteromultimers.

### ALMT transmembrane α-helices differentially contribute to channel activity

**Figure 5:**
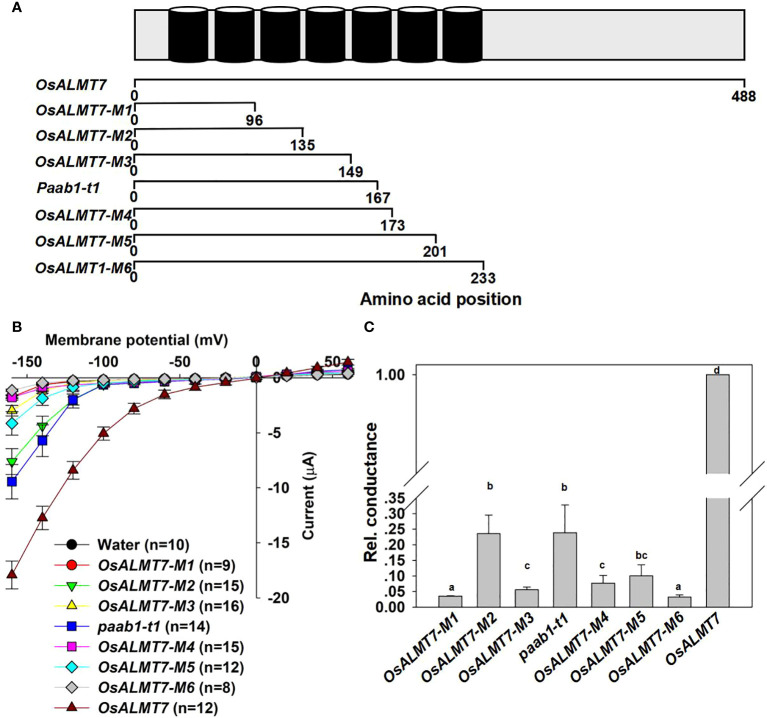
Other truncate mutants of ALMTs show channel activity and inhibit channel activity of wild-type channels. **(A)** Diagram illustrating the secondary structure of OsALMT7 with 7 transmembrane helices (upper row, modified from Ligaba et al., 2013 and referring to Heng *eta/.* (2018) and the diagram illustrating the truncate mutants of OsALMT?. OsALMT7-M1: 1-96 amino acid, with 2 transmembrane helices; OsALMT7-M2: 1-135 amino acid, with 3 transmembrane helices; OsALMT7-M3: 1-149 amino acid, with 4 transmembrane helices; OsALMT7-M4: 1-173 amino acid, with 5 transmembrane helices; OsALMT7- M5: 1-201 amino acid, with 6 transmembrane helices; OsALMT7-M6: 1-233 amino acid, with 7 transmembrane helices. **(B)** Current-Voltage relationship from TEVC recordings of whole X */aevis* oocytes expressing *OsALMTl, OsALMT7-M1, OsALMT7-M2, OsALMT7-M3, paab1-t1, OsALMT7-M4, OsALMT7- M5, OsALMT7-M6*, and water injected control with 46 nol f 200 mM Na 2-malate preloaded. The data are presented as mean± SE. (n12 for each data). **(C)** Rei. cconductance of *OsALMTl* (set as 1) and different *OsALMTl* truncate mutants cRNA injected oocytes. Different letters represent significant differences (p < 0.05, one-way ANOVA).

The *paab1* mutant terminates transcription in the middle of the 5^th^ transmembrane α-helices, causing a lack of the last 2 transmembrane α-helices and C-terminal cytosolic domains (**Figure 1A**, Heng et al., 2018). Truncated mutants of OsALMT7 with different numbers of transmembrane α-helices were constructed to examine their contribution to channel activity in the context of the whole protein (**Figure 5A**). These truncations were named *OsALMT7-M1* to *OsALMT7-M6* and contained 2 to 7 transmembrane α-helices respectively. Surprisingly, we found that OsALMT7-M2 with just 3 transmembrane α-helices mediated malate efflux, while OsALMT7-M6 with all 7 helices showed no channel activity (**Figures 5B, C** and **Supplemental Figure 3**).

**Figure 6:**
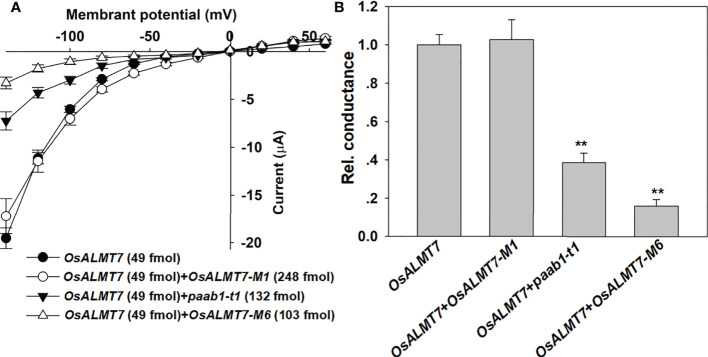
other truncate mutants of ALMTs inhibit channel activity of wild-type channels. **(A)** 1-V relationship from TEVC recordings of whole X */aevis* oocytes injecting *OsALMTl, OsALMT7+0sALMT7- M1, OsALMT7+paab1-t1*, and *OsALMT7+0sALMT7-M6* with 46 nol f 200 mM Na 2-malate pro-loaded. The truncated mutants were illustrated in **Figure 2A**, and the data are presented as mean± SE. (n 12 for each data). **(B)** Rei. conductance of *OsALMTl, OsALMT7+0sALMT7-M1, OsALMT7+paab1-t1*, and *OsALMT7+0sALMT7-M6* cRNA injected oocytes. Conductance of OsALMT7 was set as 1, and student's t test (**P<0.01) was used to analyse statistical significance from *OsALMTl*.

To investigate other truncated mutant could form heteromer with OsALMT7, we detected the inhibitory effects of OsALMT7-M1 and OsALMT7-M6 to OsALMT7 channel activity. We found that OsALMT7-M1 with 2 helices did not inhibit OsALMT7, while OsALMT7-M6 did (**Figures 6A, B**). These data confirmed that OsALMT7 functions as a multimer and found that at least 3 helices were necessary for the multimer formation.

Furthermore, we truncated TaALMT1 to *TaALMT1-M1* (1-148 amino acid residues with 3 and half transmembrane α-helices), which corresponds to *paab1-t1* in rice, *TaALMT1-M2* with 4 transmembrane α-helices, and TaALMT1-M3 with all 6 predicted transmembrane α-helices (corresponding to TaALMT1^Δ219-459^ in Ligaba et al., 2013) (**Supplemental Figure 4A**). TEVC recordings showed that, in contrast to the wild-type TaALMT1, all the truncations showed no channel activity at pH 4.5 with Al^3+^ similar to the result in Ligaba et al., 2013. However, at pH 7.2, these truncations showed malate permeability, and the current increased with the number of transmembrane α-helices increasing (**Supplemental Figures 4B, C**).

To investigate the broader implications of our observations in other ALMTs, we examined the effect of combinations between TaALMT1 and its truncated mutants. When exposed to either pH 4.5 with Al^3+^ or pH 7.2 in the bath, the truncated mutants TaALMT1-M1 and TaALMT-M3 inhibited the channel activity of TaALMT1, indicating that TaALMT1 functions as a homomultimer like OsALMT7 (**Supplemental Figures 4D, E**).

According to the studies in OsALMT7 and TaALMT1, we summarized that mediating anion flux with lacking of transmembrane helices was special for OsALMT7, and the dominant deactive proformance of its truncations to wild-type channels was common to the ALMT family as they function as dimer.

## Discussion

### ALMTs lacking their complete transmembrane α-helices can still retain the ability to transport anions

Ligaba et al. (2013) removed the C-terminal domain of TaALMT1 to various degrees and retained the full complement of transmembrane domains (TaALMT1^Δ219-459^) and found amino acid residues in C- terminal important for Al^3+^ sensitivity and the retention of malate transport ability, they also truncated transmembrane domains and found no activity of the protein. Zhang et al. (2013) found that key sites in the transmembrane helices of AtALMT9 affected channel activity. Recently, Li et al. (2020) found that truncating apple ALMT9 at the C-terminus affected channel activity. Our work is the first to show that truncating the transmembrane domains of ALMTs can still result in a transport competent protein and that such truncation results in an *in planta* phenotype (Heng et al., 2018).

We truncated different transmembrane helices of OsALMT7 and investigated the activity of them. However, only OsALMT7-M2, with 3 transmembrane helices, and paab1 showed channel activity, suggesting that the first 3 transmembrane helices are important for the pore formation on PM for malate permeability, and OsALMT7-M2 and paab1 could form a pore but other truncations with more transmembrane helices could not. Previous studies showed that the last 2 transmembrane helices were essential for pore formation and channel activity (Ligaba et al., 2013; Zhang et al., 2013), yet we found that OsALMT7-M2 and paab1 were functional for transport. These results are novel and will inform ALMT structural studies.

For TaALMT1, we got none channel activity for all the truncations at both pH 4.5 with Al^3+^ or pH 7.2. However, Ligaba et al. (2013) found that TaALMT1-M3 (TaALMT1^Δ219-459^) showed channel activity but lost Al sensitivity at pH 4.5, while our study failed to obtain TaALMT1-M3 channel activity for at least 5 times TEVC experiments. We think that this was caused by the different conditions of the *X. laevis* oocytes in the two labs.

### ALMTs function as multimers

In this study, we have shown that the PM-localized channels OsALMT7 and TaALMT1 function as multimers by TEVC recording in *X. laevis* oocytes and physical interaction analysis in tobacco leaves. Although structural biology evidence is lacking, in light of the cases of OsALMT7, we propose that it functions as multimers. Zhang et al., 2013 proposed that the vacuolar ALMT channel AtALMT9 functions as a multimer. Similar to our study, they coexpressed point mutations and wild-type AtALMT9 channels in tobacco mesophyll protoplasts, detecting the inhibition of channel activity, and they further showed the multimer formation by immunoblot analysis. Recently, Wang et al., 2022 and Qin et al., 2022 reported that ALMT1 and ALMT12 functions as a dimer. Our study started from the clew in Heng et al. (2018). when wild-type plants were transformed with a genomic fragment containing the *paab1* base substitution causing both *OsALMT7* and *paab1* expressing and resulting in a panicle abortion phenotype. That dominant-negative phenotype implied that paab1 might inhibit OsALMT7 channel activity and the following experiment approved that. Although we cannot predict the OsALMT7 forming a dimer or a trimer, it is credible that the geometric symmetry structure formed with monomer unit is necessary for the ALMT anion channels. Furthermore, we propose that introduction of mutant ALMT channels to wildtype plants would be a method to alter ALMT function as a tool to manipulate plant phenotype.

In the BiFC experiment, we detected interaction fluorescence only when YFP truncations were fused at the N-terminus of OsALMT7 or paab1 (**Figure 3**) but no fluorescence when YFP truncations were fused at the C-terminus (data not shown). One reason is that unlike TaALMT1, OsALMT7 was predicted to have 7 transmembrane helices, and the C-termini of OsALMT7 and paab1 might face different side of the PM respectively. Another is that Mumm et al. (2013) showed that fusing a YFP at the N-terminus had no effect on channel characteristics and PM localization for ALMT12, while the C-terminus fused YPF affected function and PM localization of ALMT12. So, we proposed that YFP truncations fusing in the C-terminus might inhibit the interaction between OsALMT7 and paab1. These might be the reason that the tandem construction causes the malate conductance reducing comparing to WT OsALMT7 channel (**Figure 4**). Furthermore, for the case of the tandems of paab1-t1-OsALMT7 and OsALMT7-paab1-t1, which protein was designed at the N-terminal did affect the malate permeability of the tandem. According to the TEVC data, we proposed that the paab1-t1-OsALMT7 provided more complete pore than OsALMT7-paab1-t1. Moreover, the tandem with paab1 in the N-terminus had stronger channel activity than in C-terminus (**Figure 4**) suggesting that paab1 mutants and OsALMT7 channel need a especial combination to mediate malate transporting.

## Data availability statement

The original contributions presented in the study are included in the article/**Supplementary Material**. Further inquiries can be directed to the corresponding authors.

## Author contributions

HZ and ZH (co-first author) performed the electrophysisology and BiFC experiments. YXL made the vector constructions. YL and CF(corresponding author) designed the project and wrote the manuscript. All authors contributed to the article and approved the submitted version.

## Acknowledgments

We thank Prof. Matthew Gilliham (Uni of Adelaide) for suporting the project, kindly pre-reviewing the manuscript and helping for the English writing.

## Conflict of interest

The authors declare that the research was conducted in the absence of any commercial or financial relationships that could be construed as a potential conflict of interest.

## Publisher’s note

All claims expressed in this article are solely those of the authors and do not necessarily represent those of their affiliated organizations, or those of the publisher, the editors and the reviewers. Any product that may be evaluated in this article, or claim that may be made by its manufacturer, is not guaranteed or endorsed by the publisher.
