# Supplementary figures and images for: Multiple ALMT subunits combine to form functional anion channels: A case study for rice ALMT7
Source: Front Plant Sci. 2022 Nov 14;13:1012578. doi: 10.3389/fpls.2022.1012578 (PMC9702572; doi:10.3389/fpls.2022.1012578)

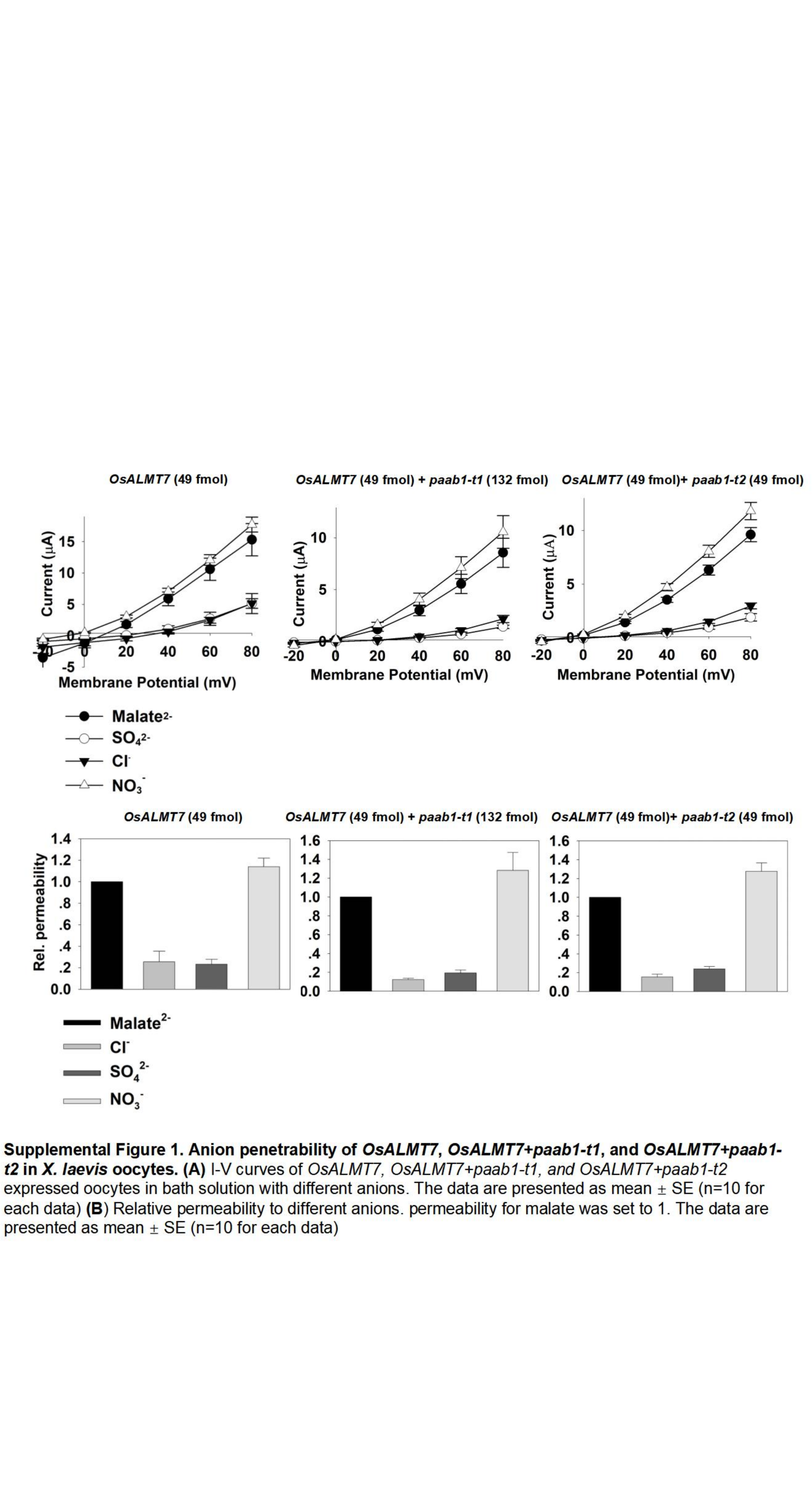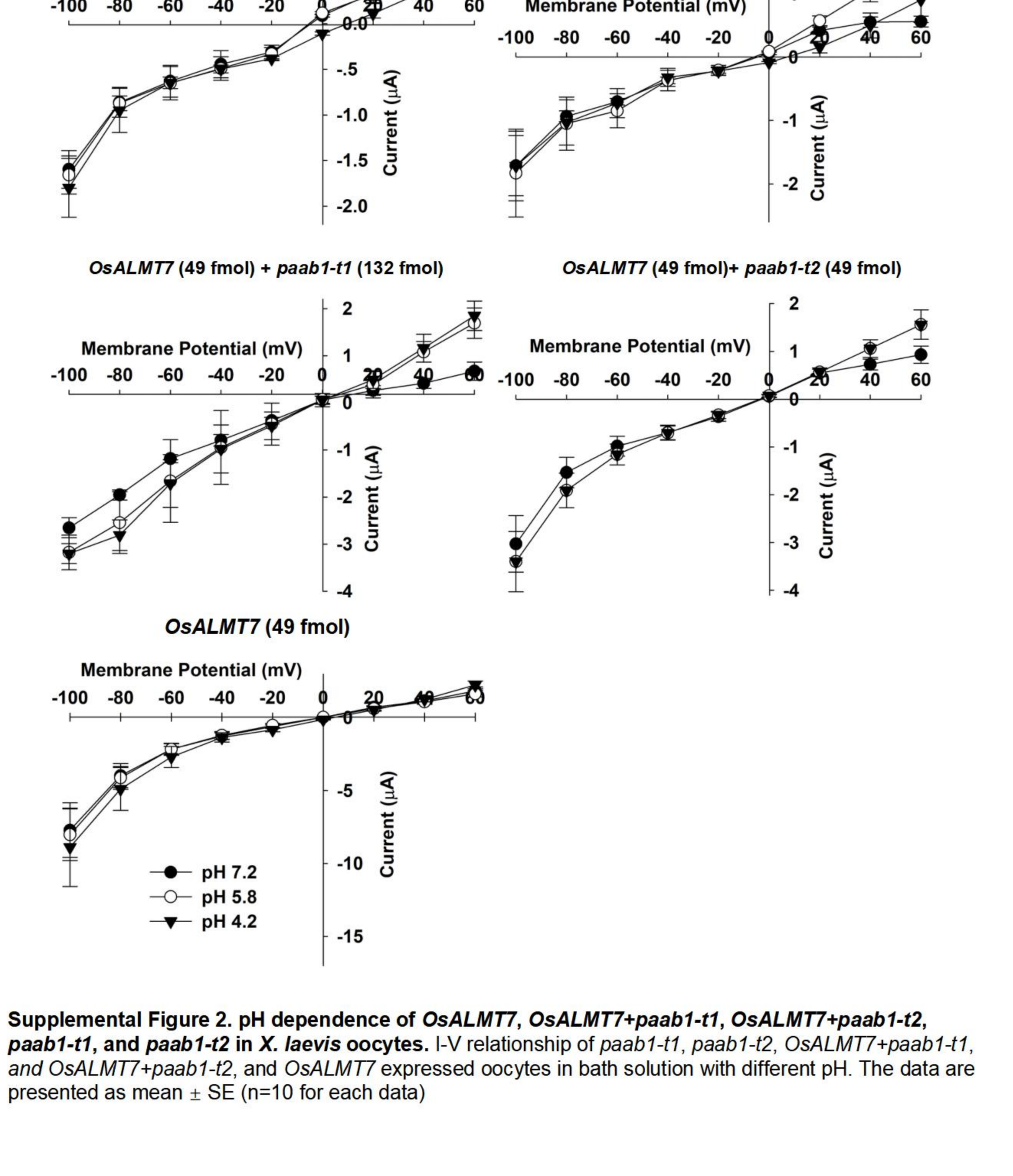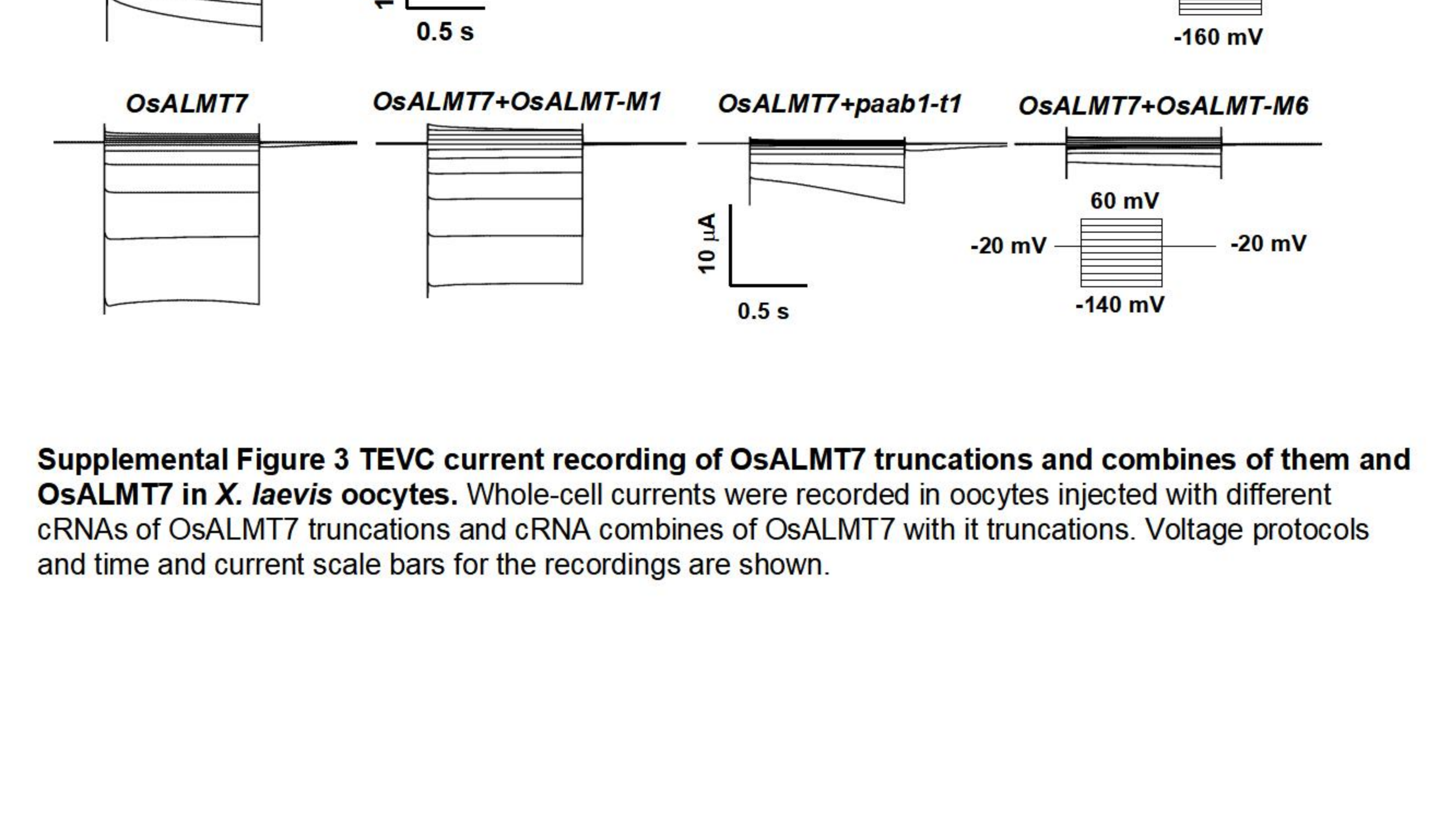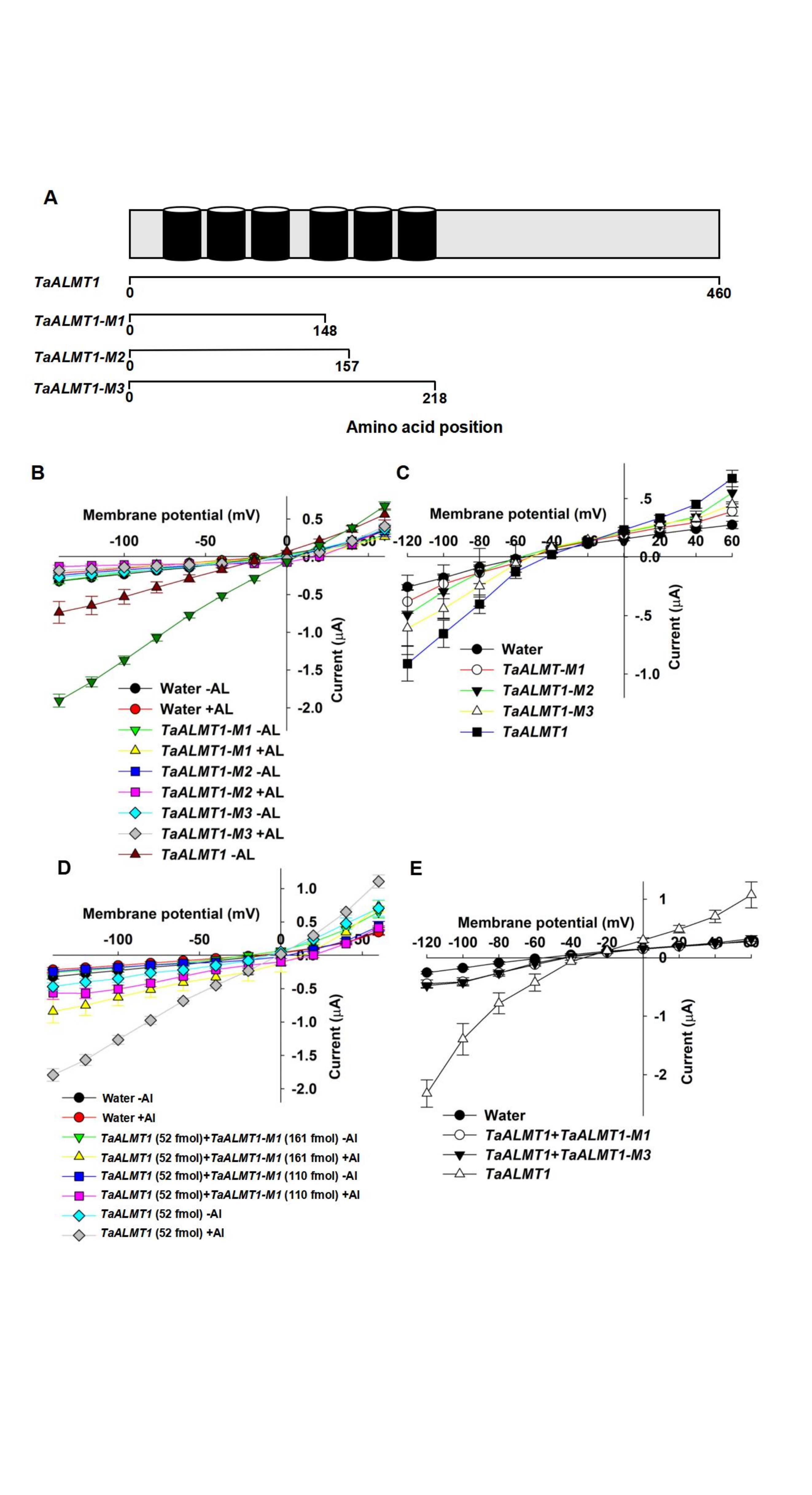

Supplement: Supplementary file 1 [file DataSheet_1.pdf]
